# Exciton tuning in monolayer WSe_2_*via* substrate induced electron doping†

**DOI:** 10.1039/d2na00495j

**Published:** 2022-10-25

**Authors:** Yang Pan, Mahfujur Rahaman, Lu He, Ilya Milekhin, Gopinath Manoharan, Muhammad Awais Aslam, Thomas Blaudeck, Andreas Willert, Aleksandar Matković, Teresa I. Madeira, Dietrich R. T. Zahn

**Affiliations:** Semiconductor Physics, Institute of Physics, Chemnitz University of Technology Chemnitz Germany yang.pan@physik.tu-chemnitz.de; Center for Materials, Architectures, and Integration of Nanomembranes (MAIN), Chemnitz University of Technology Chemnitz Germany; Department of Electrical and Systems Engineering, University of Pennsylvania Philadelphia PA USA; Center for Microtechnologies, Chemnitz University of Technology Chemnitz Germany; Institute of Physics, Montanuniversität Leoben Leoben Austria; Fraunhofer Institute for Electronic Nano Systems Chemnitz Germany

## Abstract

We report large exciton tuning in WSe_2_ monolayers *via* substrate induced non-degenerate doping. We observe a redshift of ∼62 meV for the A exciton together with a 1–2 orders of magnitude photoluminescence (PL) quenching when the monolayer WSe_2_ is brought in contact with highly oriented pyrolytic graphite (HOPG) compared to dielectric substrates such as hBN and SiO_2_. As the evidence of doping from HOPG to WSe_2_, a drastic increase of the intensity ratio of trions to neutral excitons was observed. Using a systematic PL and Kelvin probe force microscopy (KPFM) investigation on WSe_2_/HOPG, WSe_2_/hBN, and WSe_2_/graphene, we conclude that this unique excitonic behavior is induced by electron doping from the substrate. Our results propose a simple yet efficient way for exciton tuning in monolayer WSe_2_, which plays a central role in the fundamental understanding and further device development.

## Abstract 2

We report large exciton energy tuning (∼62 meV) in WSe_2_ monolayers *via* substrate induced non-degenerate electron doping.

## Graphical abstract

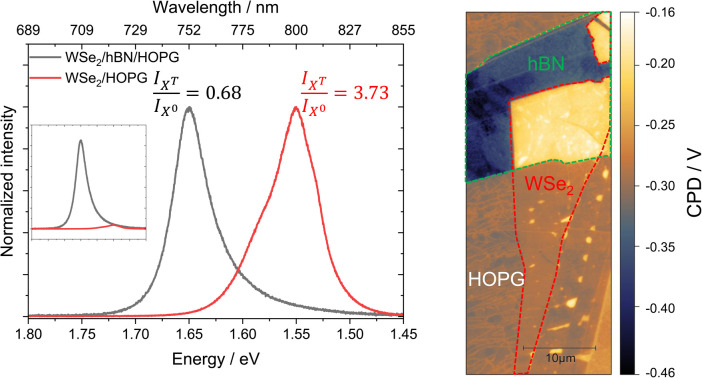

## 1 Introduction

Beyond graphene,^1^ transition metal dichalcogenides (TMDCs) are currently at the center of 2D materials research, owing to their extraordinary optical, electrical, thermal, and mechanical properties,^2–5^ and, most importantly, to the unique indirect- to direct-bandgap transition when the material is thinned from bulk to monolayer.^6,7^ The direct bandgap nature of monolayer TMDCs makes them promising materials for next generation optoelectronic devices.^8–10^ Exciton tuning and bandgap engineering become extremely important in this case, as they build the basis for fundamental research including but not limited to exciton–polariton, many-body physics, and optical selection rules.^11–15^ Moreover, they are the key for sufficiently widening the application field and devices towards 2D photonics and optoelectronics.^16,17^ Different approaches of exciton tuning and bandgap engineering have been reported such as changing the dielectric environment, mechanical straining, doping, alloying, injecting plasmonic hot electrons, and manipulating the carrier concentration *via* an external electric field.^18–25^

In this work, we report tuning the exciton energy in monolayer WSe_2_*via* substrate induced non-degenerate electron doping. We observe an ∼62 meV redshift for the A exciton (from ∼1.65 eV to ∼1.59 eV) together with a few orders of magnitude photoluminescence (PL) quenching when the monolayer WSe_2_ is brought in contact with HOPG compared to the WSe_2_ excitonic feature on dielectric substrates such as hBN, SiO_2_, and polydimethylsiloxane (PDMS), which has been measured and reported in our previous work.^26^ As a by-product, a drastic increase of the intensity ratio of trions to neutral excitons up to 5.5 times was observed, which is a characteristic of electron doping in TMDC monolayers. To understand this unique behavior, we employed a systematic PL and Kelvin probe force microscopy (KPFM) investigation on different TMDC/substrate combinations, namely WSe_2_/HOPG, WSe_2_/graphene, and WSe_2_/hBN as a reference. Surprisingly, we were only able to observe such a pronounced redshift when WSe_2_ was in contact with HOPG but not with graphene. The KPFM measurements provide different contact potential difference (CPD) values when comparing WSe_2_/HOPG to WSe_2_/graphene and WSe_2_/hBN. This indicates different Fermi level positions and different carrier concentrations in WSe_2_. The PL quenching, redshift, increase of the intensity ratio of trions to neutral excitons, and different CPD values all conclusively point towards electrons from the HOPG substrate injected to WSe_2_ and leading to bandgap renormalization and thus the tuning of exciton energy. Our work explains the unique behavior of monolayer WSe_2_/HOPG and demonstrates a simple yet efficient method, which enables the exciton energy in monolayer WSe_2_ to be tuned by ∼62 meV. This is essential for fundamental studies and the development of devices such as photodetectors, excitonic LEDs, and coupling with plasmonics.^10,27–30^

## 2 Materials and methods

### 2.1 Sample preparation

**Fig. 1:**
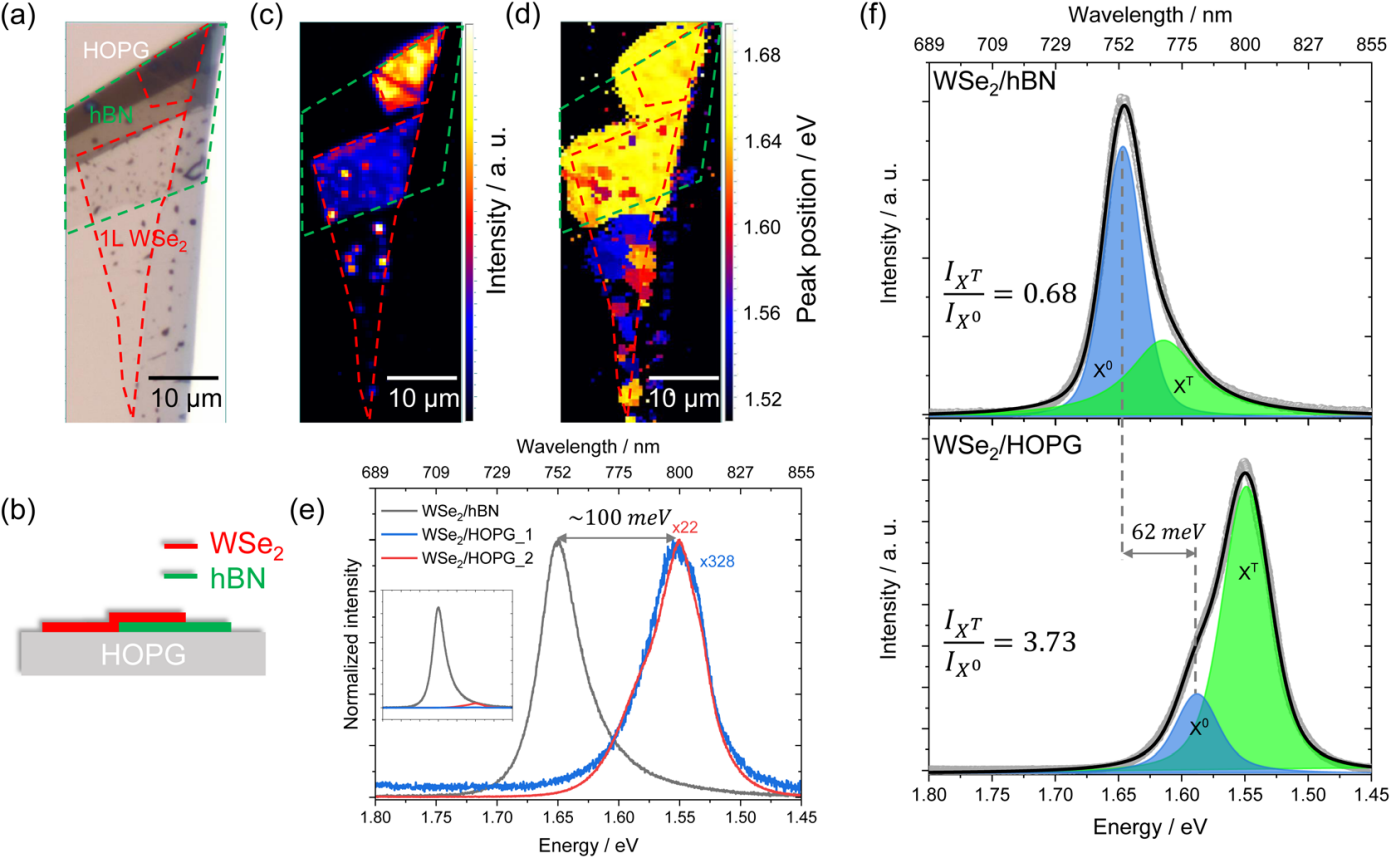
(a) Optical microscopy image and (b) schematic cross-section of the WSe_2_/hBN/HOPG hetero-stack. PL (c) intensity and (d) peak position map of the sample. (e) PL spectra of WSe_2_/hBN and WSe_2_/HOPG. For comparison, the PL intensity of WSe_2_/HOPG is normalized to that of WSe_2_/hBN. Inset: as-measured (not-normalized) PL spectra. (f) Fitted PL spectra of WSe_2_/hBN and WSe_2_/HOPG.

**Fig. 2:**
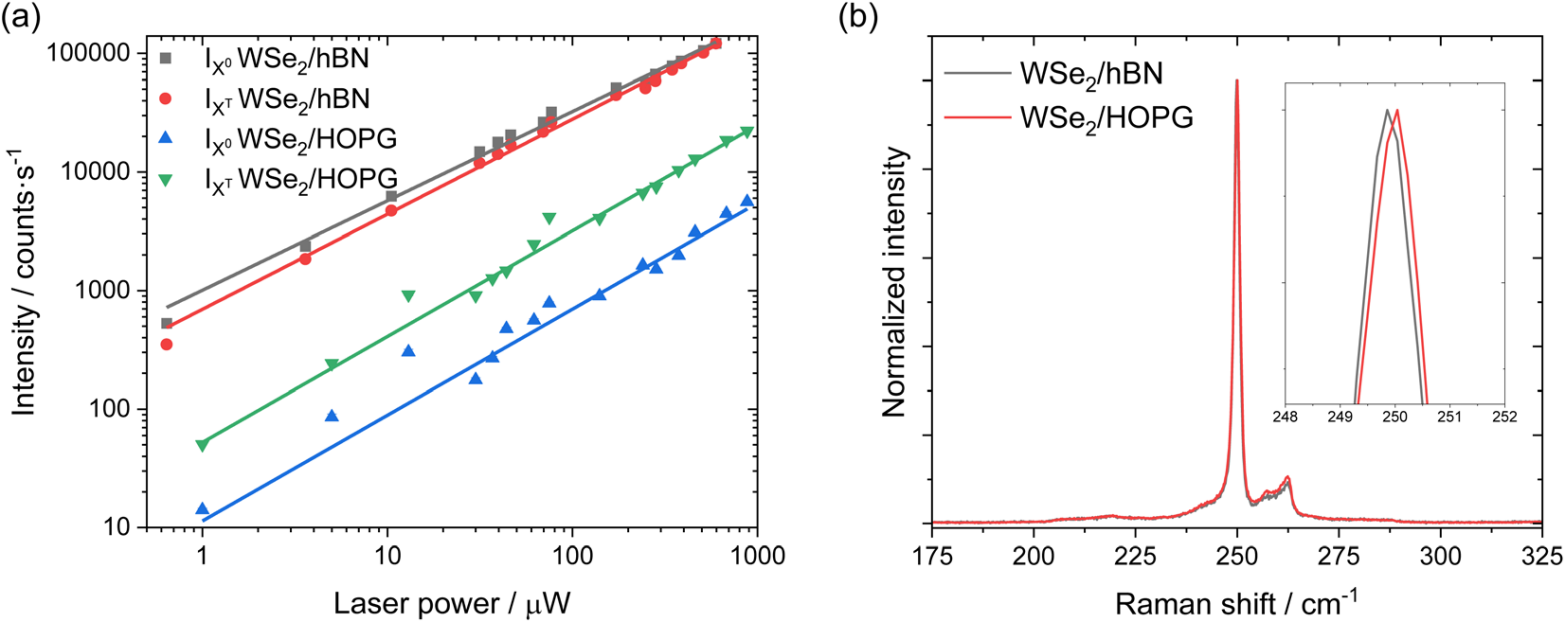
(a) PL intensity as a function of excitation power for *X*^0^ and *X*^T^ emissions from WSe_2_/hBN and WSe_2_/HOPG. Solid lines are fits to a power law: *I* ∝ *P*^*α*^. (b) High spectral resolution Raman spectra of WSe_2_/hBN and WSe_2_/HOPG. Inset is a zoom in at 248–252 cm^−1^.

Few layer hBN (from 2D semiconductors), graphene (from NGS Naturgraphit), and monolayer WSe_2_ (from HQ graphene) are mechanically exfoliated from their bulk crystals *via* Nitto tape onto a PDMS stamp and then transferred bottom-to-top onto the HOPG substrate following a deterministic all-dry transfer technique.^31,32^ All materials on PDMS are first characterized by PL and Raman prior to transfer. HOPG is cleaved before transfer to ensure a fresh surface. After transfer, the samples are annealed in a nitrogen atmosphere at 150 °C for 2 hours to optimize the contact between flakes and ensure a clean surface. The detailed process used for sample fabrication is shown in Fig. 1S and 2S.†

### 2.2 Optical spectroscopy

PL measurements are performed using a Horiba Xplora Plus equipped with a 100×, 0.9 NA objective, a spectrometer comprising a 600 l mm^−1^ grating, and an electron-multiplying CCD (EMCCD). A DPSS 532 nm CW laser source was used for excitation. The laser power is ∼100 μW measured under the objective for PL measurements if not specified differently. The setup is equipped with a Märzhäuser motorized *xyz* stage with a 100 nm step size precision for PL mapping.

Raman spectra are acquired using a Horiba LabRAM HR spectrometer with a 100×, 0.9 NA objective, a 2400 l mm^−1^ grating, and a liquid nitrogen cooled Symphony CCD detector. A solid-state 514.7 nm laser is used for excitation with a laser power of ∼100 μW measured under the objective. We choose a confocal pinhole of 50 μm to reach a high spectral resolution of approximately 0.8 cm^−1^.

### 2.3 Kelvin probe force microscopy

We use an AIST-NT SmartSPM™ 1000 for KPFM measurements. The KPFM measurements are performed under ambient conditions with constant temperature and humidity. The NSG10 Pt coated tip is commercially available with a typical tip radius of ∼35 nm.

## 3 Results and discussion

Fig. 1(a) and (b) display the optical microscopy image and the schematic cross-section of a WSe_2_/hBN/HOPG hetero-stack, respectively. The monolayer WSe_2_ is transferred onto the hBN/HOPG hetero-stack in a way that it creates contacts with both few layer hBN and HOPG. According to the atomic force microscopy (AFM) measurement shown in Fig. 8S,† the top brown-colored hBN has a thickness of ∼38.2 nm, and the middle part has a thickness of ∼3.8 nm. We acquired a micro PL map on the sample with a step size of 0.5 μm. As shown in the intensity map in Fig. 1(c), the PL intensity of WSe_2_ on thick hBN is higher than that on thin hBN because of interference enhancement.^33,34^ More importantly, comparing the PL intensity of WSe_2_ on hBN and HOPG, one can clearly identify that a drastic decrease of PL intensity occurs on HOPG. Several mechanisms can cause the low PL quantum yield and thus lead to the decrease of PL intensity, such as high defect concentration, strong many-body effects, charge dissociation and charge transfer.^34–37^ Since the samples investigated in this work are prepared by mechanical exfoliation from the same flake, we attribute the PL intensity decrease to charge dissociation or charge transfer. The few dots that still remain intense may correspond to bubbles or hydrocarbon contaminants at the interface, which can enhance the PL signal (more information about the influence of bubbles on the PL spectra can be found in Fig. 5S†).^38–40^Fig. 1(d) displays the peak position map indicating that the sample is clearly divided into two parts: WSe_2_/hBN with a peak position of ∼1.65 eV and WSe_2_/HOPG with a peak position of ∼1.55 eV.

**Fig. 3:**
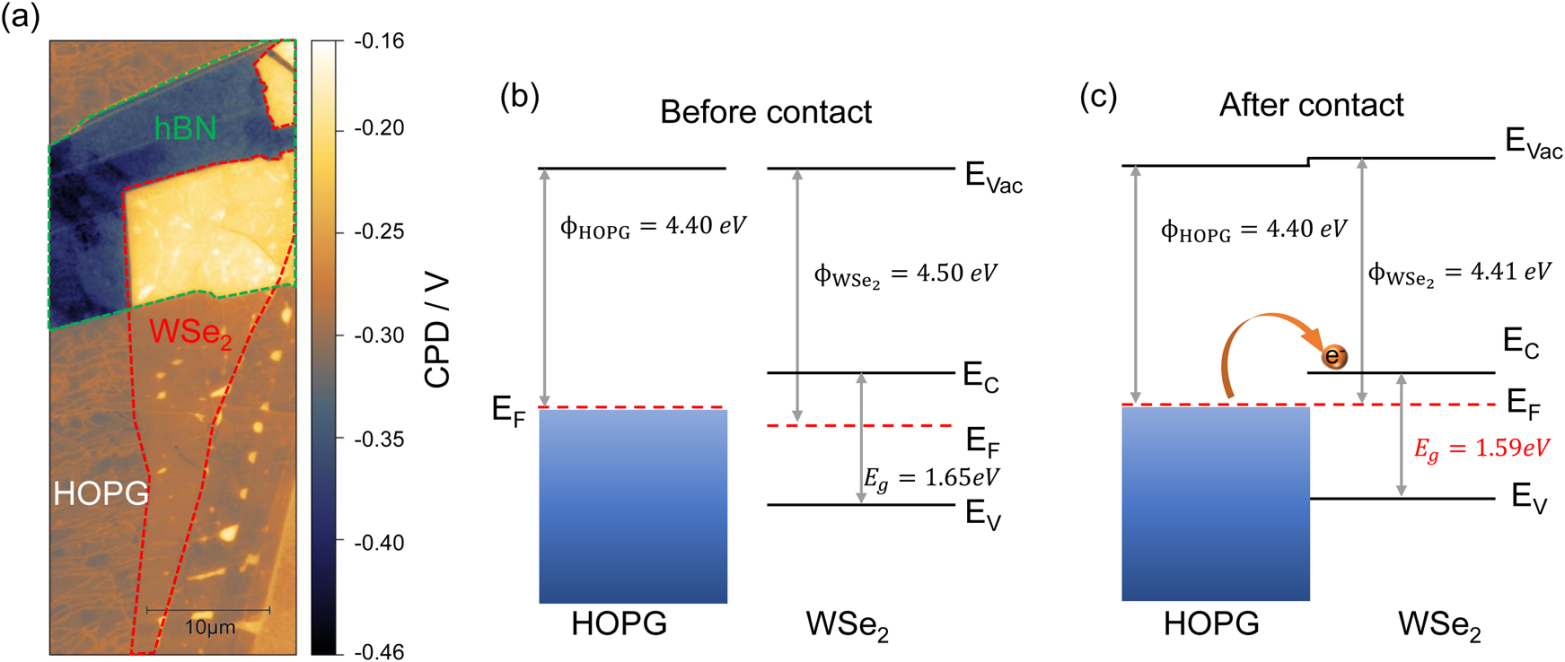
(a) KPFM of WSe_2_/hBN/HOPG. Band diagram of monolayer WSe_2_ and HOPG before (b) and after (c) contact. Before contact means when WSe_2_ is isolated from HOPG by hBN and after contact means that WSe_2_ is on HOPG.

The detailed spectra of WSe_2_/hBN and WSe_2_/HOPG are shown in Fig. 1(e). A strong PL quenching of 1–2 orders of magnitude is observed when WSe_2_ is in contact with HOPG, which indicates charge dissociation through the junction or charge transfer between WSe_2_ and HOPG.^34,35^ Monolayer WSe_2_ on hBN shows a characteristic PL at ∼1.65 eV, which is consistent with the literature values,^26,41^ while the PL peak position of WSe_2_/HOPG shows a marked ∼100 meV redshift, which is much higher than the reported value caused by changing of the dielectric environment.^18^ Besides the quenching and redshift, the PL line shape changes significantly. We thus deconvoluted the PL spectra into peaks corresponding to the radiative recombination of different exciton/trion species in monolayer WSe_2_ (detailed fitting parameters can be found in the ESI†). As shown in Fig. 1(f), two peaks with a Voigt line shape were identified in the fitted spectra. The neutral exciton (*X*^0^) originates from the direct bandgap transition at the *K* point in the Brillouin zone and there is a charged exciton peak also known as trion *X*^T^.^41–44^ We also investigated the Stokes shift of monolayer WSe_2_ as shown in Fig. 3S,† which is negligible with a value of ∼2 meV. It is therefore fair enough to consider the PL peak position as the exciton energy. The fitting result suggests a 62 meV redshift of *X*^0^ and most interestingly, a drastic increase of the relative *X*^T^ intensity. The ratio of *I*_*X*^T^_/*I*_*X*^0^_ increases from 0.68 ± 0.01 on hBN to 3.73 ± 0.04 on HOPG, which is strong evidence of a higher electron concentration in WSe_2_ on HOPG than in WSe_2_ on hBN.

Even though we propose that charge transfer and electron doping from HOPG to monolayer WSe_2_ seem to be the most reasonable mechanism of PL quenching, redshift, and increasing intensity ratio of trions to neutral excitons, we still carefully examined whether they originate from the defect-bound localized states or strain due to lattice mismatch. Power dependent PL intensities of WSe_2_/hBN and WSe_2_/HOPG are displayed in Fig. 2(a). The PL intensity is obtained from the integrated area of the Voigt fitted *X*^0^ and *X*^T^ features. The PL intensity as a function of excitation laser power is then fitted by a power law: *I* ∝ *P*^*α*^,^41,45^ where the extracted exponential factor 
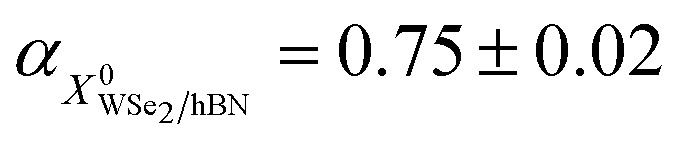
, 
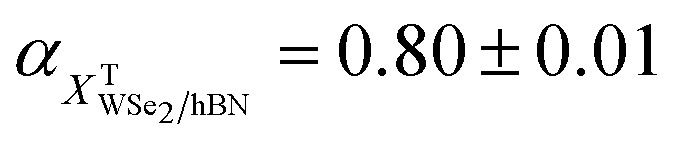
, 
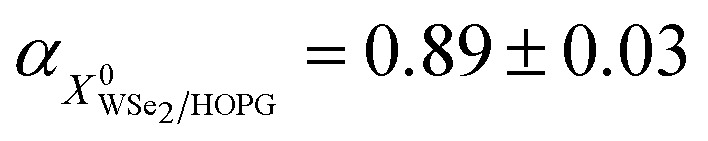
, and 
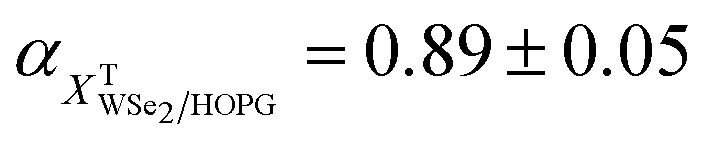
 for *X*^0^ and *X*^T^ on WSe_2_/hBN and WSe_2_/HOPG, respectively. The fitting results suggest a sublinear power dependence of the PL intensity for both *X*^0^ and *X*^T^ on WSe_2_/hBN and WSe_2_/HOPG and do not show any saturation phenomena at high laser power, which excludes the possibility of defects as the origin of the observed behavior.^46^Fig. 2(b) shows the high spectral resolution (∼0.8 cm^−1^) Raman spectra of WSe_2_/hBN and WSe_2_/HOPG (only two spectra are shown in the main text for clarity, more spectra can be found in the ESI†). The most intense peak at ∼250 cm^−1^ corresponds to the combination of the in-plane E_2g_ and out-of-plane A_1g_ vibrational modes, which are almost degenerate at the same frequency.^26,47–49^ The feature at ∼260 cm^−1^ is a second order peak caused by a double resonance effect involving the longitudinal acoustic phonon at the *M* point in the Brillouin zone assigned as 2LA(*M*).^49,50^ The E_2g_/A_1g_ mode is highly sensitive to the strain.^51,52^ The fitted Raman spectra reveal a small 0.15 cm^−1^ peak position difference, which indicates that strain is also small and cannot account for the huge redshift in PL.

KPFM is a powerful technique to obtain the local surface potential and Fermi level position in the nanoscale.^34,53^ We therefore measured KPFM on the WSe_2_/hBN/HOPG hetero-stack to obtain further insight into the energy level alignment at the various interfaces. In the ideal case KPFM measures the contact potential difference (CPD) between the metallic AFM tip and the sample according to the relation: CPD = (*ϕ*_sample_ − *ϕ*_tip_)/*e*, where *ϕ*_sample_ and *ϕ*_tip_ are the work functions of the sample and the tip, and *e* is the elementary charge. KPFM does not give a quantitative, absolute value of the Fermi level position under ambient conditions, because the CPD value is known to be strongly influenced by the measurement environment, tip geometry, and parasitic effects such as capacitive coupling, as well as the chosen experimental parameters.^54–57^ Nevertheless, it still qualitatively indicates the trend of the Fermi level position and material work functions.^34,53,58,59^ The values of the energy levels discussed in the following paragraph are directly extracted from the KPFM measurements.

Fig. 3(a) shows the CPD map of WSe_2_/hBN/HOPG. Even though it is the same monolayer WSe_2_ flake, one can clearly distinguish the high contrast between WSe_2_ on hBN and WSe_2_ on HOPG. The absolute work function of HOPG is determined to be *ϕ*_HOPG_ = 4.4 eV by the ultraviolet photoelectron spectroscopy (UPS) measurement shown in Fig. 10S.† The electron affinity of monolayer WSe_2_ is reported to be 3.7–3.9 eV.^60,61^ We therefore calculate and draw the band diagrams of WSe_2_ before (on hBN) and after (on HOPG) coming into contact with HOPG as shown in Fig. 3(b) and (c), respectively. The band diagrams reveal a decrease of the work function or increase of the Fermi level when WSe_2_ is in contact with HOPG, which indicates a higher electron concentration in WSe_2_ on HOPG than in WSe_2_ on hBN. The high electron concentration in WSe_2_/HOPG can only originate from electron doping from HOPG to WSe_2_, which explains the PL quenching, redshift, and increasing intensity ratio of trions to neutral excitons shown in Fig. 1.

**Fig. 4:**
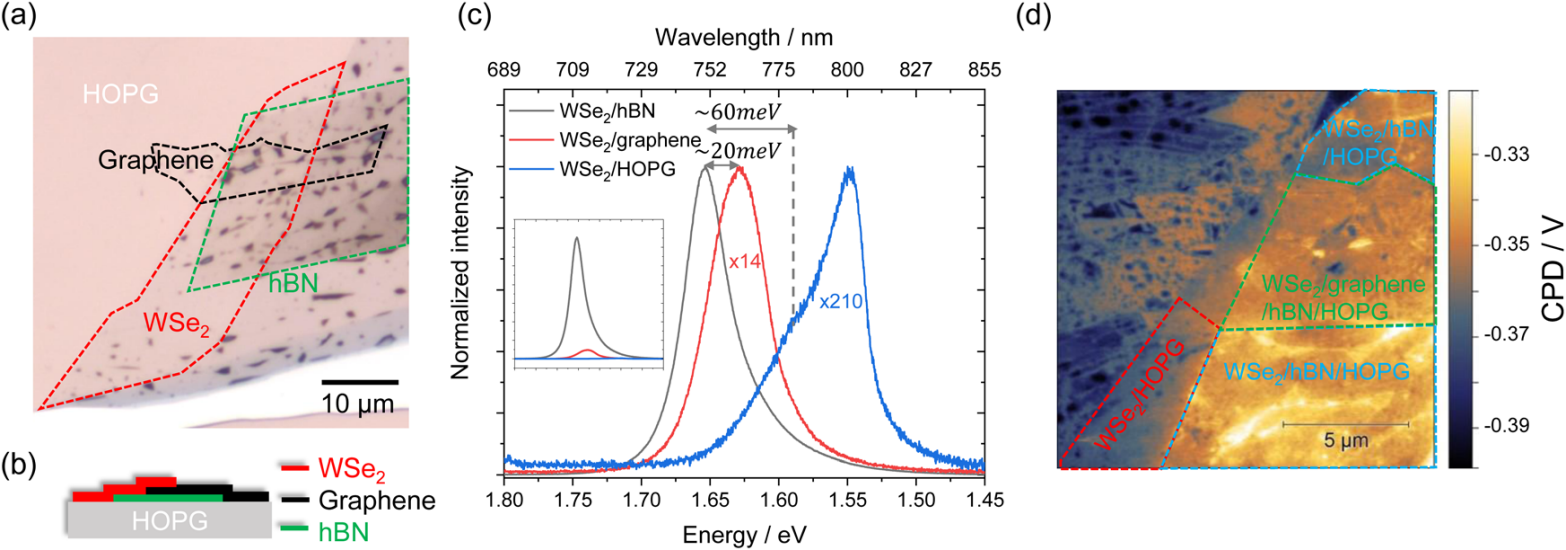
(a) Optical microscopy image and (b) schematic cross-section of the WSe_2_/graphene/hBN/HOPG hetero-stack. (c) PL spectra of WSe_2_/hBN, WSe_2_/graphene, and WSe_2_/HOPG. For comparison, the intensities of the WSe_2_/graphene and WSe_2_/HOPG PL are normalized to that of WSe_2_/hBN/HOPG. Inset: as-measured (not-normalized) PL spectra. (d) KPFM of WSe_2_/graphene/hBN/HOPG.

Apparently interfacing WSe_2_ with HOPG results in efficient tuning of the exciton emission in a straightforward manner. Researchers also studied the combination of WSe_2_ and graphene,^18^ yet did not report similar results. This naturally leads to the question: do graphene and graphite lead to a different interaction when interfaced with WSe_2_? To answer this question, we prepared a hetero-stack of WSe_2_/graphene/hBN/HOPG as shown in Fig. 4(a) and (b), where WSe_2_ is partially on hBN, partially on graphene, and partially on HOPG. The PL spectra of WSe_2_/hBN, WSe_2_/graphene, and WSe_2_/HOPG are shown in Fig. 4(c). Again, we observe a similar PL quenching, redshift, and increasing intensity ratio of trions to neutral excitons for WSe_2_ on HOPG. However, a redshift of only 20 meV is detected on WSe_2_/graphene, which is in excellent agreement with the value reported by Raja *et al.*^18^ This redshift of the A exciton is attributed to the altered local dielectric screening of the Coulomb interaction in WSe_2_. Besides the 20 meV redshift, there is a clear broadening of the PL for WSe_2_ on graphene compared with that of WSe_2_ on hBN. We consider that this broadening originates from the environmental dielectric disorder introduced by the several stamping steps during the sample preparation or the charge transfer between WSe_2_ and graphene.^62,63^ A higher trion emission intensity is also not observed in the case of WSe_2_/graphene. This clearly indicates that the interaction of WSe_2_ on graphene is different from that of WSe_2_ on HOPG. We assume that this difference is due to the lower amount of free electrons in graphene than that in HOPG. The KPFM measurement performed on such a sample is displayed in Fig. 4(d). A CPD contrast is only observed between WSe_2_/HOPG and WSe_2_/hBN with a value of ΔCPD_WSe_2_/hBN−WSe_2_/HOPG_ = (20.4 ± 4.9) mV, while WSe_2_/graphene and WSe_2_/hBN reveal a negligible difference of ΔCPD_WSe_2_/hBN−WSe_2_/gr_ = (2.4 ± 4.3) mV. This suggests that a significant change of the Fermi level position occurs due to electron doping from the substrate and only happens for WSe_2_ on HOPG but not for WSe_2_ on graphene. We use the mass action model to estimate the carrier concentration in the monolayers (details in the ESI†).^64,65^ Assuming that the radiative decay rates of excitons and trions are in the same order of magnitude as described in ref. ^64^, the electron concentration is in the range of ∼10^13^ cm^−2^ in monolayer WSe_2_ interfaced with HOPG, while it is approximately one magnitude smaller when interfaced with hBN or graphene.

## 4 Conclusions

In summary, we investigated WSe_2_/hBN, WSe_2_/graphene, and WSe_2_/HOPG hetero-stacks. We observed a strong PL intensity quenching, 62 meV redshift of the A exciton, and a drastic increase of the intensity ratio of trions to neutral excitons on WSe_2_/HOPG compared to WSe_2_/graphene and WSe_2_/hBN. The KPFM results reveal a high CPD contrast, which indicates a renormalization of the energy level alignment at the interface. The effects observed for WSe_2_ on HOPG are thus assigned to significant electron doping of the WSe_2_ monolayer from the HOPG substrate. We propose a simple yet efficient way to tune the exciton emission in monolayer WSe_2_ by substrate induced electron doping.

## Author contributions

Y. P. fabricated the samples, performed the measurements and analyzed the data. M. R., I. M. L. H., and T. I. M. contributed to data analysis and discussion. G. M., T. B. and A. W. performed the reflectance contrast measurement. M. A. A. and A. M. provided the graphene. D. R. T. Z. supervised the work. M. R. and D. R. T. Z. were involved in the evaluation and interpretation of the results. Y. P. wrote the manuscript. All authors discussed the results and commented on the manuscript.

## Conflicts of interest

The authors declare no conflict of interest.

## Supplementary Material

NA-004-D2NA00495J-s001
