# Supplementary material for: Exciton tuning in monolayer WSe2via substrate induced electron doping
Source: Nanoscale Adv. 2022 Oct 25;4(23):5102–8. doi: 10.1039/d2na00495j (PMC9680939; doi:10.1039/d2na00495j)
Supplement: NA-004-D2NA00495J-s001 [file NA-004-D2NA00495J-s001.pdf]

Electronic Supplementary Material (ESI) for Nanoscale Advances.
This journal is © The Royal Society of Chemistry 2022

Supplementary information: Exciton tuning in monolayer WSe_2 via substrate induced electron doping

Yang Pan[1,2*], Mahfujur Rahaman[3], Lu He[1,2], Ilya Milekhin[1,2], Gopinath Manoharan[4], Muhammad Awais Aslam[5], Thomas Blaudeck[2,4,6], Andreas Willert[6], Aleksandar Matković[5], Teresa I. Madeira[1,2], and Dietrich R. T. Zahn[1,2]

[1]Semiconductor Physics, Institute of Physics, Chemnitz University of Technology, Chemnitz, Germany
[2]Center for Materials, Architectures, and Integration of Nanomembranes (MAIN), Chemnitz University of Technology, Chemnitz, Germany
[3]Department of Electrical and Systems Engineering, University of Pennsylvania, Philadelphia, PA, USA
[4]Center for Microtechnologies, Chemnitz University of Technology, Chemnitz, Germany
[5]Institute of Physics, Montanuniversität Leoben, Leoben, Austria
[6]Fraunhofer Insitute for Electronic Nano Systems, Chemnitz, Germany
[*]Corresponding author: yang.pan@physik.tu-chemnitz.de

1 Sample preparation

1.1 WSe_2/hBN/HOPG hetero-stack

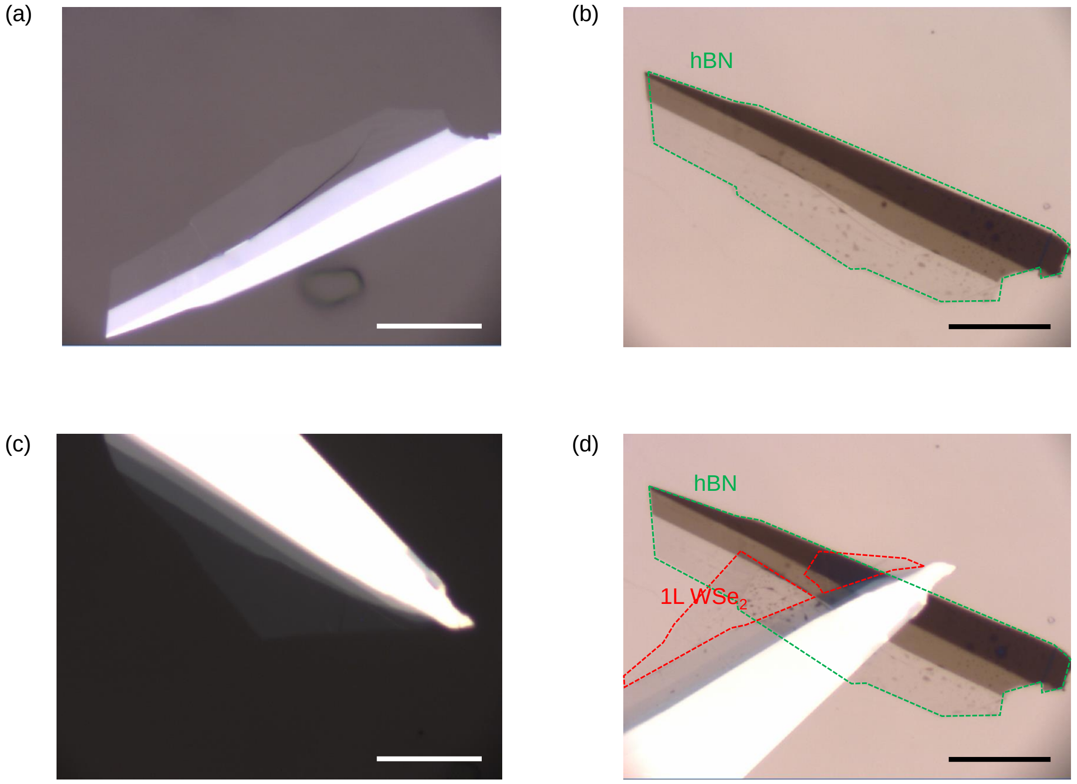

Figure 1S: Optical microscope images of few layer hBN on (a) PDMS, (b) HOPG and monolayer WSe_2 on (c) PDMS, (d) hBN/HOPG. Scale bar in figure is 20 µm.

Monolayer WSe_2 and few layer hBN are mechanical exfoliated from their bulk materials via Nitto tape on PDMS stamp (as shown in Fig. 1S (a) and (c)). WSe_2 is firstly characterized by PL and Raman spectroscopy to identify the layer numbers before transfer. After confirming the layer numbers, the HOPG top layer is cleaved to ensure a clean surface. The hBN and WSe_2 are immediately transferred bottom-to-top with a all-dry deterministic transfer technique[1].

1.2 WSe_2/graphene/hBN/HOPG hetero-stack

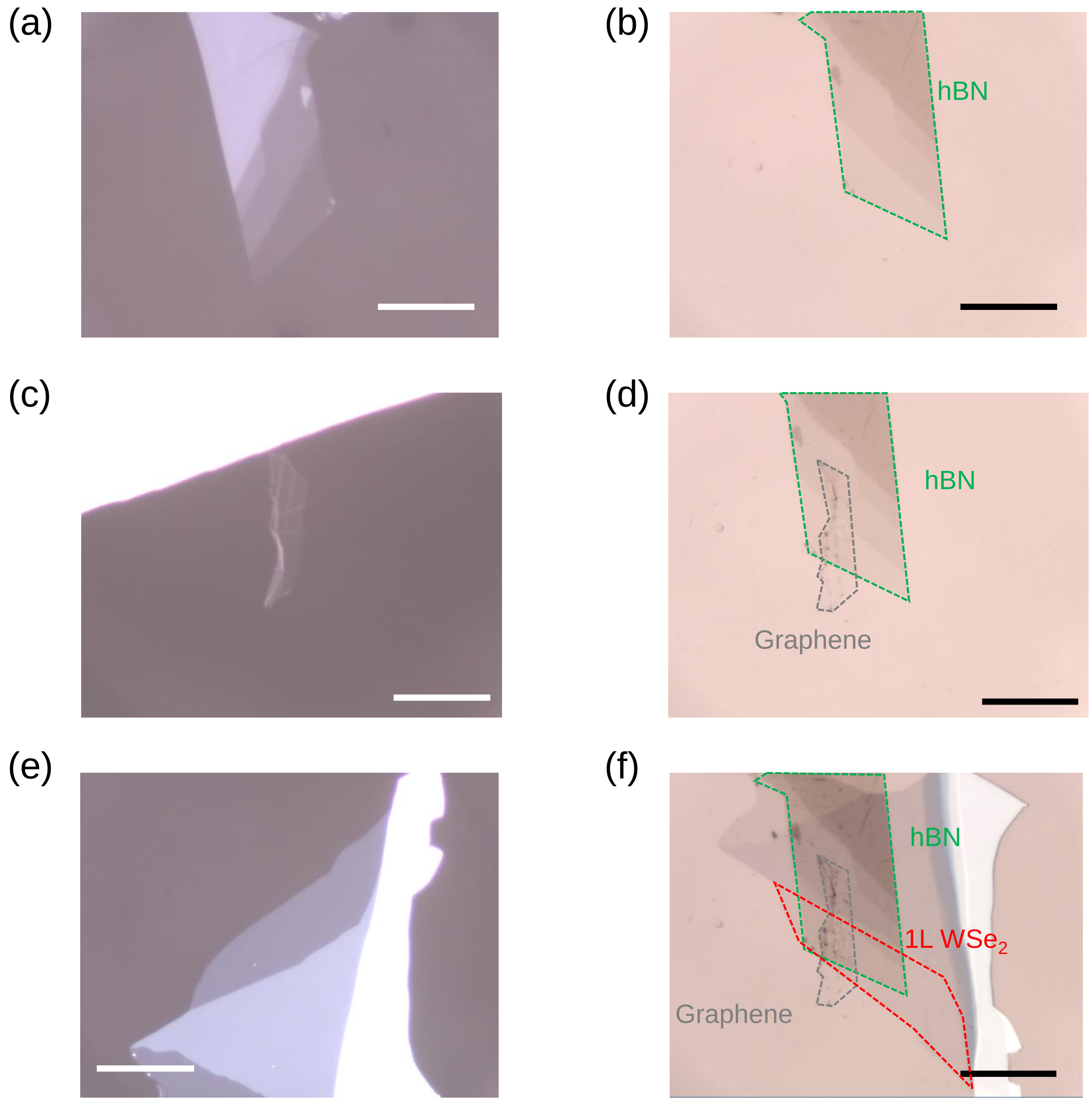

Figure 2S: Optical microscope images of few layer hBN on (a) PDMS, (b) HOPG, graphene on (c) PDMS, (d) hBN/HOPG and monolayer WSe_2 on (e) PDMS, (f) graphene/hBN/HOPG. Scale bar in figure is 20 µm.

The sample preparation procedure is same as mentioned above.

2 Stokes shift of monolayer WSe_2

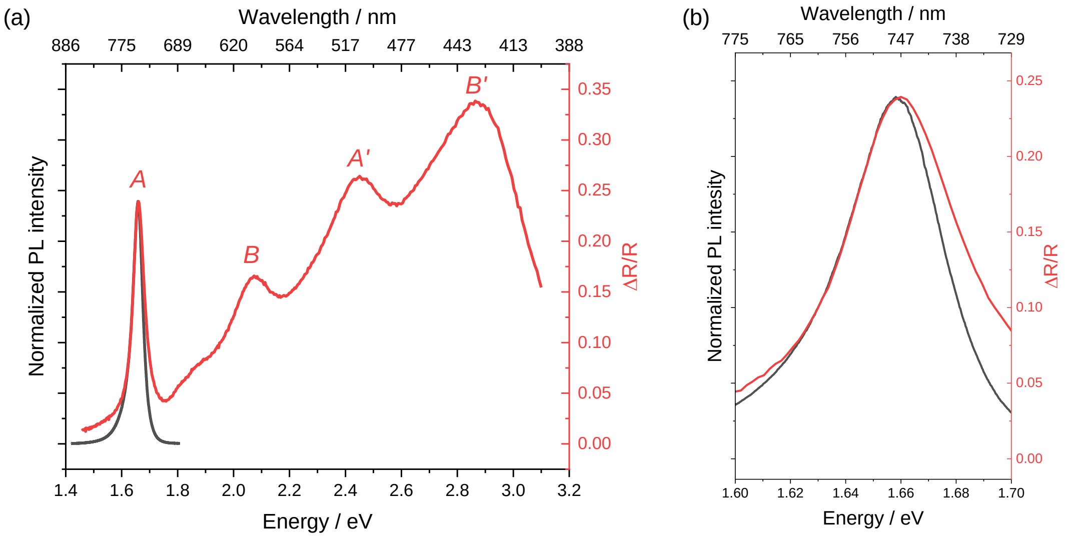

Figure 3S: (a) Micro PL and micro reflectance contrast spectra of monolayer WSe_2. (b) zoomed in for 1.60-1.70 eV.

Micro reflectance contrast measurements are carried out with a Zeiss AxioImager.M2m microscope in epi-illumination configuration equipped with a 50x, 0.75NA objective, a Zeiss HAL 100 illuminator-12 V/ 100W white-light source with intensity control and coupled to a J&M Analytik AG Tidas S MSP 800 spectrometer operable in the spectral range 200-980 nm [2, 3].

For the ultra-thin film on a transparent substrates, $\Delta R/R$ is predominantly determined by the imaginary part of the dielectric function, which is proportional to the optical absorption[4–7].

We measured the micro PL and micro reflectance contrast spectra to extract the Stokes shift of monolayer WSe_2 to make sure that it is reasonable to consider the PL peak energy position corresponding to the exciton energy. As shown in Fig. 3S, we only observe a ~2 meV Stokes shift, which makes it fair enough to consider the exciton PL peak position as the exciton energy.

3 Extended information of the PL maps

3.1 Fitting parameters for the PL spectrum

WSe_2/hBN/HOPG	Position / meV	FWHM / meV
X^0	1647±2	37±2
X^T	1615±2	57±2

WSe_2/HOPG	Position / meV	FWHM / meV
X^0	1585±2	42±2
X^T	1549±2	46±2

Note that the errors from fitting are less than 1 meV, which is well below the measuring limit at room temperature. We measured a Hg-Ar calibration lamp to determine our instrumental broadening and considered the instrumental broadening as the error.

3.2 Histogram of the WSe_2/HOPG/hBN PL map

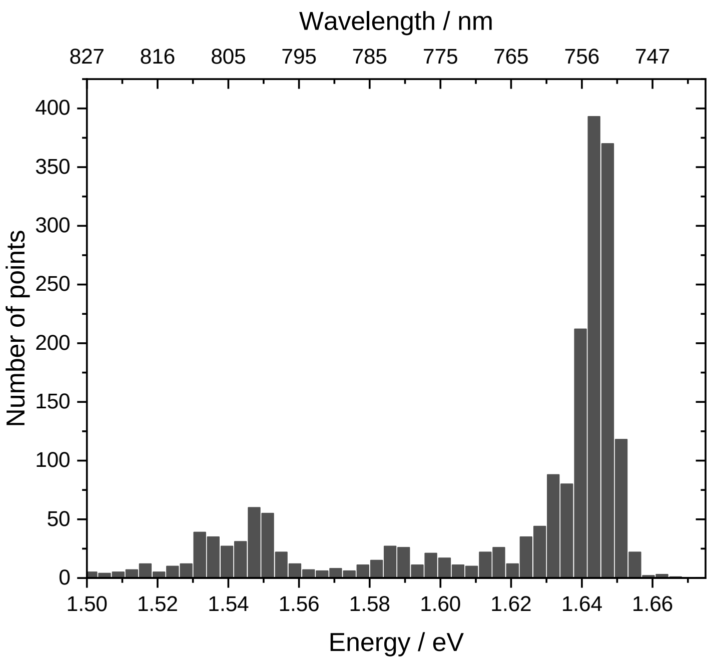

Figure 4S: Histogram of the WSe_2/hBN/HOPG PL peak position map in figure. 1(b) in the main text.

3.3 Effect of bubbles on the PL spectra

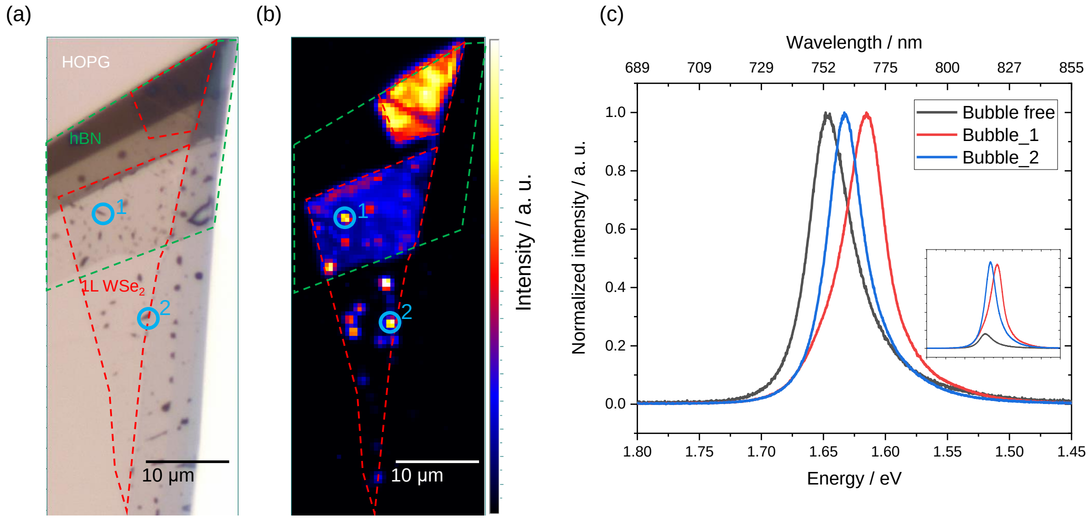

Figure 5S: (a) optical microscope image and (b) PL intensity map of the WSe_2/hBN/HOPG heterostack. (c) Typical PL spectra of the monolayer WSe_2 with and without bubble. Inset: as-measured (not-normalized) PL spectra.

3.4 PL map of WSe_2/graphene/hBN/HOPG hetero-stack

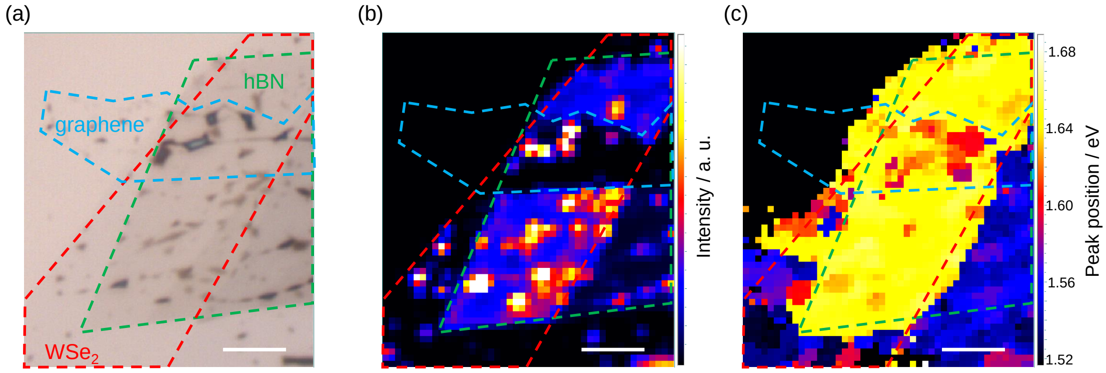

Figure 6S: (a) optical microscope image, (b) PL intensity map and (c) PL peak position map of the WSe_2/graphene/hBN/HOPG hetero-stack. Scale bar in figure is 5 µm.

4 High-resolution Raman spectra

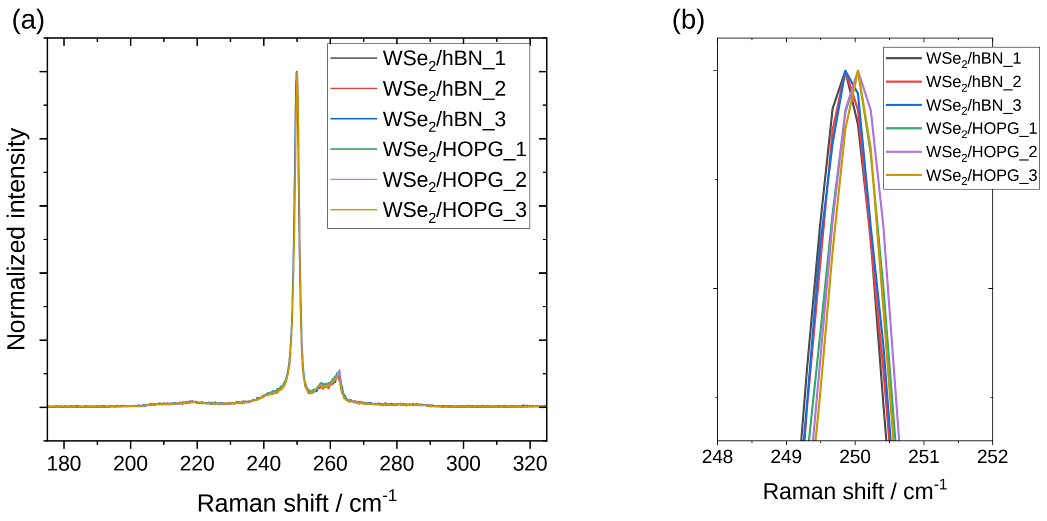

Figure 7S: (a) High-resolution Raman spectra of WSe_2/hBN and WSe_2/HOPG. (b) Zoomed in for 248-252 cm^{-1}.

5 AFM and KPFM

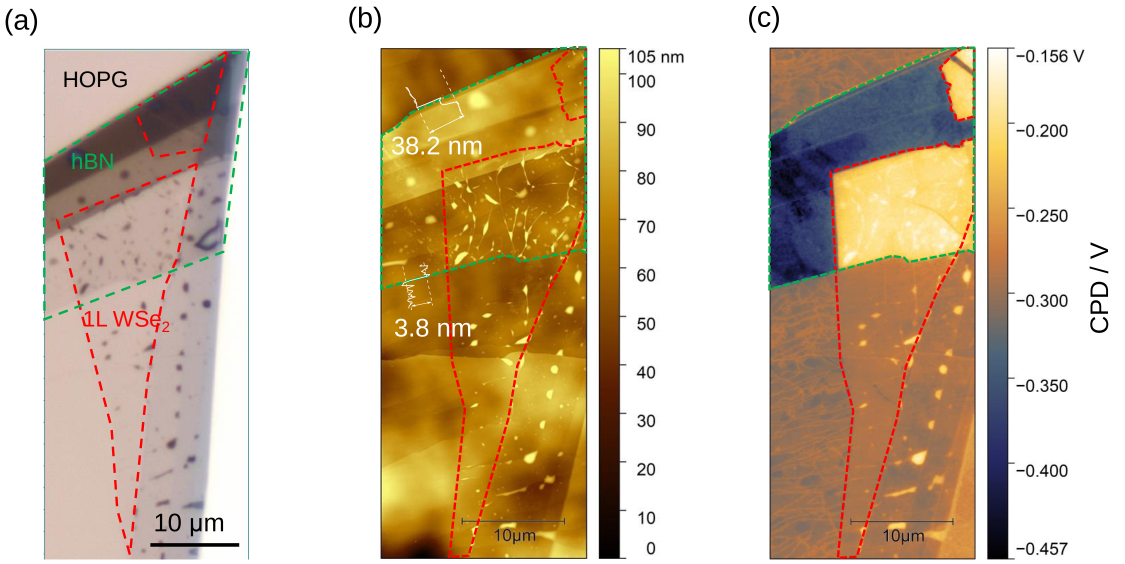

Figure 8S: (a) optical microscope image, (b) AFM height image, and (c) KPFM image of WSe_2/hBN/HOPG hetero-stack.

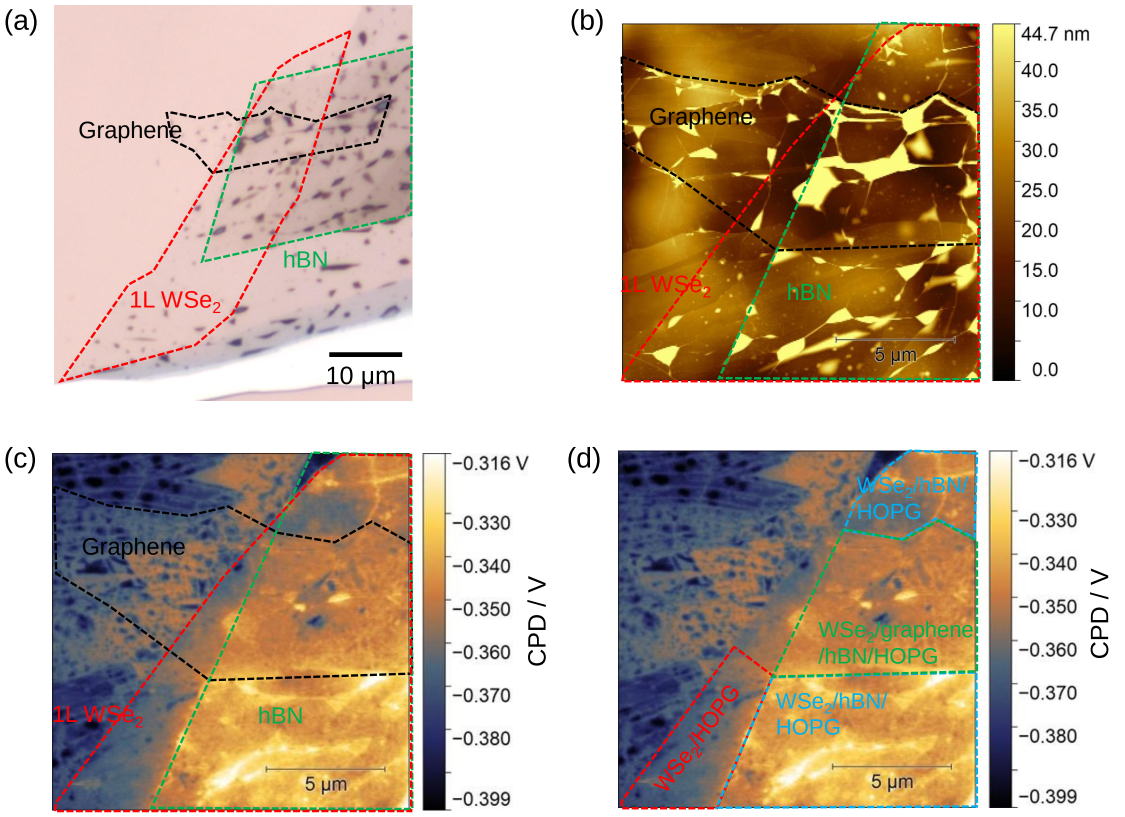

Figure 9S: (a) optical microscope image, (b) AFM height image, and (c-d) KPFM image of WSe_2/graphene/hBN/HOPG hetero-stack.

6 Work function determination of HOPG

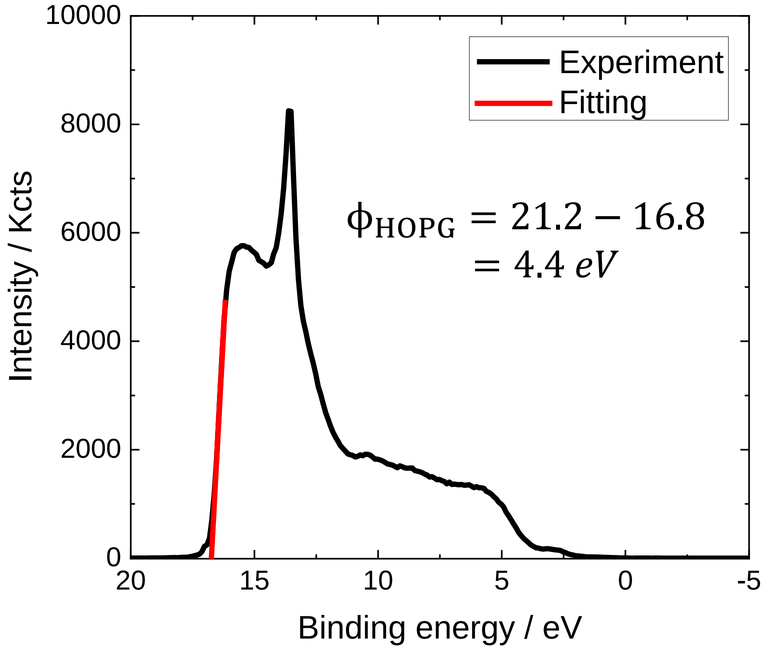

Figure 10S: UPS spectra of HOPG.

We use ultraviolet photoelectron spectroscopy (UPS) to determine the absolute work function of HOPG [8]. The He-I light source has an energy of 21.2 eV and the secondary electron cutoff (SEC) is 16.8 eV. The work function of HOPG is 4.4 eV.

7 Mass action model

We estimate the electron concentration using the mass action law associated with trions [9, 10]. In this model the following relation is obtained:

$$\frac{N_X n_e}{N_{X^-}} = \left(\frac{4 m_X m_e}{\pi \hbar^2 m_{X^-}}\right) k_B T exp\left(-\frac{E_b}{k_B T}\right) \tag{1}$$

where N_X and N_{X^-} are the exciton and trion population, respectively. n_e is the electron concentration. m_e ($0.48 m_0$), m_X ($0.92 m_0$), and m_{X^-} ($1.40 m_0$) are the effective mass of electron, exciton, and trion, respectively [11]. E_b (30 meV) is the trion biding energy. The intensity ration of trion to neutral exciton is expressed as:

$$\frac{I_{X^-}}{I_X} = \frac{\gamma_{X^-}}{\gamma_X} \times \frac{N_{X^-}}{N_X} = \frac{\gamma_{X^-}}{\gamma_X} \times \frac{n_e}{\left(\frac{4 m_X m_e}{\pi \hbar^2 m_{X^-}}\right) k_B T exp\left(-\frac{E_b}{k_B T}\right)} \tag{2}$$

where γ_{X^-} and γ_X are the radiative decay rate of trion and exciton. We assume the value of $\frac{\gamma_{X^-}}{\gamma_X}$ is in the same order of magnitude as what has been reported in Ref. [9], the estimated electron concentration is in the order of 10^{13} cm^{-2} when WSe_2 is interfaced with HOPG and is approximately one magnitude smaller when interfaced with hBN or graphene.

References

1. Castellanos-Gomez, A. *et al.* Deterministic transfer of two-dimensional materials by all-dry viscoelastic stamping. *2D Materials* **1,** 011002 (2014).
2. Sowade, E., Blaudeck, T. & Baumann, R. R. Self-assembly of spherical colloidal photonic crystals inside inkjet-printed droplets. *Crystal Growth & Design* **16,** 1017–1026 (2016).
3. Kuhn, E. *et al.* Disorder explains dual-band reflection spectrum in spherical colloidal photonic supraparticle assemblies. *Nano Select* **2,** 2461–2472 (2021).
4. Raja, A. *et al.* Coulomb engineering of the bandgap and excitons in two-dimensional materials. *Nature communications* **8,** 1–7 (2017).
5. Li, Y. *et al.* Measurement of the optical dielectric function of monolayer transition-metal dichalcogenides: MoS_2, $MoSe_2$, WS_2, and WSe_2. *Physical Review B* **90,** 205422 (2014).
6. Zhao, W. *et al.* Evolution of electronic structure in atomically thin sheets of WS_2 and WSe_2. *ACS nano* **7,** 791–797 (2013).
7. McIntyre, J. & Aspnes, D. E. Differential reflection spectroscopy of very thin surface films. *Surface Science* **24,** 417–434 (1971).
8. Kim, J. W. & Kim, A. Absolute work function measurement by using photoelectron spectroscopy. *Current Applied Physics* **31,** 52–59 (2021).
9. Mouri, S., Miyauchi, Y. & Matsuda, K. Tunable photoluminescence of monolayer MoS2 via chemical doping. *Nano letters* **13,** 5944–5948 (2013).
10. Ross, J. S. *et al.* Electrical control of neutral and charged excitons in a monolayer semiconductor. *Nature communications* **4,** 1–6 (2013).
11. Rasmussen, F. A. & Thygesen, K. S. Computational 2D materials database: electronic structure of transition-metal dichalcogenides and oxides. *The Journal of Physical Chemistry C* **119,** 13169–13183 (2015).
